# Prevalence of *tet*(X4) in *Escherichia coli* From Duck Farms in Southeast China

**DOI:** 10.3389/fmicb.2021.716393

**Published:** 2021-08-23

**Authors:** Yang Yu, Chao-Yue Cui, Xu Kuang, Chong Chen, Min-Ge Wang, Xiao-Ping Liao, Jian Sun, Ya-Hong Liu

**Affiliations:** ^1^Guangdong Provincial Key Laboratory of Veterinary Pharmaceutics Development and Safety Evaluation, South China Agricultural University, Guangzhou, China; ^2^National Risk Assessment Laboratory for Antimicrobial Resistance of Animal Original Bacteria, South China Agricultural University, Guangzhou, China; ^3^Joint International Research Laboratory of Agriculture and Agri-Product Safety, Institutes of Agricultural Science and Technology Development, Yangzhou University, Yangzhou, China

**Keywords:** antimicrobial resistance (AMR), *tet*(X4), ducks, feces, the environment

## Abstract

**Objectives:**

Carbapenems, colistin, and tigecycline are critically important antibiotics in clinics. After the global appearance of *bla*_*NDM*_ and *mcr* mediating the resistance to carbapenems and colistin, respectively, tigecycline becomes the last-resort drug against severe human infections caused by multidrug-resistant bacteria. Recently, a mobile tigecycline resistance gene *tet*(X4) has been identified in *Escherichia coli*, *Klebsiella pneumoniae*, and *Acinetobacter baumannii* that causes high resistance to tigecycline and other tetracyclines. In this study, the prevalence of *tet*(X4) in *E. coli* isolates from duck and goose farms in Southeast China was identified and characterized.

**Methods:**

Feces, soil, sewage, and dust samples were collected from duck and goose farms along with the southeast coast provinces of China. Antimicrobial susceptibility testing and polymerase chain reaction screening were performed to investigate the phenotype and genotype of tigecycline resistance. Conjugation, S1 pulsed-field gel electrophoresis (PFGE), and whole-genome sequencing were used to determine the transferability, genetic location, and the genomic characteristics of *tet*(X4).

**Results:**

In total, 1,716 samples were collected, and 16 isolates (0.9%) recovered from Guangdong, Shandong, and Jiangsu were positive for *tet*(X4) gene with tigecycline minimum inhibitory concentrations ≥16 mg/L. Notably, among these *tet*(X4)-positive *E. coil* isolates, seven of them were from the environment samples (soil and sewage). PFGE and multilocus sequence typing demonstrated that ST3997 was the most prevalent sequence type (eight isolates, 50%) in Jiangsu province. By conjugation assays, 11 isolates were able to transfer *tet*(X4) plasmid to *E. coli* C600 recipient, and these plasmids belonged to IncHI1 and IncX1 detected by sequence analysis. *tet*(X4) was found adjacent to an insertion sequence IS*CR2* downstream and a *cat*D gene upstream for all isolates. In addition, multiple-drug resistance to tigecycline, chlortetracycline, ampicillin, florfenicol, ciprofloxacin, gentamicin, trimethoprim/sulfamethoxazole, and fosfomycin was profiled in most of the *tet*(X4)-positive isolates.

**Conclusion:**

The identification of *tet*(X4) harboring *E. coli* strains in duck farms and their surrounding environment enlarges our knowledge of the variety and prevalence of tigecycline resistance. The prevalence of *tet*(X4) raises concern for the use of tetracyclines in animal farming, and the *tet*(X4) gene should be listed as primary gene for resistance surveillance.

## Introduction

Carbapenems, colistin, and tigecycline are considered as the last-resort antibiotics against severe human infections caused by multidrug-resistant (MDR) bacteria. However, the discovery of a series of carbapenems resistance genes, such as *bla*_*OXA*_, *bla*_*NDM*_, *bla*_*VIM*_, and *bla*_*KPC*_, as well as the mobile colistin resistance *mcr* gene, has compromised the effectiveness of carbapenems and colistin in clinics (Gangcuangco et al., 2016; Lunha et al., 2016; Wang et al., 2017; Gaibani et al., 2020). Under such circumstances, tigecycline was considered as the last chance for treatment of extensively drug-resistant pathogens. Unfortunately, the effectiveness of tigecycline was compromised by the new plasmid-borne variants of the *tet*(X) family genes *tet*(X3) and *tet*(X4) that mediate the resistance to tigecycline, as well as to the newly approved eravacycline and omadacycline (He et al., 2019). The identification of tigecycline resistance has again raised challenges for clinical treatment of critical infections, especially caused by carbapenem-resistant Enterobacteriaceae (CRE) in public health.

The tetracycline family of therapeutic agents has been in commercial use since 1940s, but the increasing incidence of bacterial resistance has relegated older tetracyclines to a limited role for treating common infectious diseases (Villano et al., 2016). Three new tetracyclines generations (tigecycline, omadacycline, and eravacycline) have been discovered that circumvent the common tetracycline resistance mechanisms. Tigecycline, a glycylcycline tetracycline, has demonstrated antibacterial activity across a broad spectrum of Gram-positive, Gram-negative, anaerobic, and atypical bacteria (Peterson, 2008). It was approved for complicated skin and intra-abdominal infections by both the Food and Drug Administration (FDA) in the United States and the European Medicines Agency (European medicines agency, 2013). It can be applied for the community-acquired bacterial pneumonia approved by FDA as well (FDA, 2005). Tigecycline exhibits antimicrobial susceptibility against broad-spectrum pathogens including the CRE and even colistin-meropenem–coresistant *Escherichia coli* (Yu et al., 2019).

However, tigecycline resistance has been reported to be mediated by different resistant mechanisms. Evolution of the *tet*A gene decreases tigecycline susceptibility with minimum inhibitory concentrations (MICs) from 1 rising to 32 mg/L and leads to treatment failure in carbapenem-resistant *Klebsiella pneumoniae* infections (Du et al., 2018). Overexpression of the resistance–nodulation–cell division–type efflux system plays a major role in tigecycline resistance in clinical *Acinetobacter nosocomialis* (Yang et al., 2019), and the mutations at the tip of the extended loop of the ribosomal S10 protein have been associated with tigecycline resistance in different bacterial species (Angeles Argudin et al., 2018). The latest identification of the plasmid-mediated high-level tigecycline resistance gene, *tet*(X4), has again decreased the promised prospect of using tigecycline in the clinic (He et al., 2019; Sun et al., 2019a). For treating common community- and hospital-acquired infections, omadacycline, a first-in-class aminomethylcycline antibiotic, has been newly approved by FDA and is active against extended-spectrum beta-lactamase–producing bacteria, methicillin-resistant *Staphylococcus aureus*, and even vancomycin-resistant enterococcus (Macone et al., 2014). Eravacycline, also approved by FDA, is a fully synthetic fluorocycline antibiotic and active against clinically important pathogens mostly resistant to cephalosporins, fluoroquinolones, beta-lactams, and carbapenems (Zhanel et al., 2016). The severely important aspect of the *tet*(X4) gene is that it also causes the resistance to both omadacycline and eravacycline (Sun et al., 2019a).

Aquaculture is a diversified production sector with different production systems and practices. Although centralization-breeding factory are increasingly popular in China, one of the integrated farming systems (duck or goose–fish production) still plays a significant role in southeast China (Miao, 2010). In this study, we investigated the prevalence of *tet*(X4) in duck and goose farms belonging to different breeding patterns in Southeast China. Isolation ratio for *tet*(X4) gene was analyzed, and the genomic profiling was conducted for a comprehensive understanding of the genomic background of *tet*(X4) gene. Furthermore, conjugation assays were tested to determine the dissemination and transferability of the *tet*(X4) gene.

## Materials and Methods

### Sampling Information and Bacterial Strains

**FIGURE 1:**
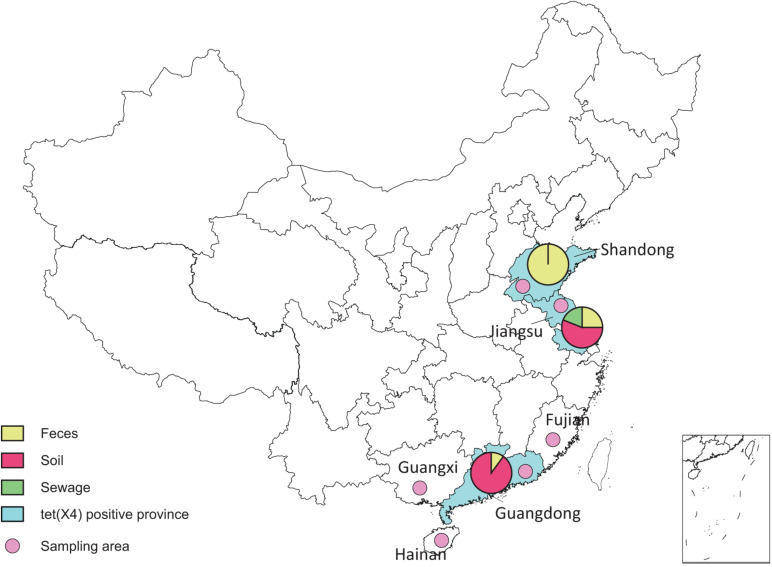
Sampling areas of duck and goose farms in southeast China.

From May 1, 2017, to January 1, 2019, we collected 1,716 consecutive, non-duplicate samples, including fecal (1211), soil (259), sewage (228), and dust (18) samples, from 25 duck farms and 3 goose farms in six provinces (Shandong, Jiangsu, Fujian, Guangdong, Hainan, and Guangxi) along with the southeast coast of China (Figure 1 and Supplementary Table 1). All duck and goose farms investigated in this study were divided into three different breeding patterns (Supplementary Figure 1): (A) on filter net and shelves, (B) along the river without shed, and (C) duck–fish production system. Feces samples were collected freshly from the dropping trays (breeding pattern A) or the grounds (B and C), and dust samples were scrubbed from the windows or doors of sheds in patterns A and C. The fecal samples were randomly collected from ducks and geese, with approximately 60 samples per farm. The dust samples were collected with cotton swab and transferred into the 2-mL centrifuge tubes with normal saline. Soil samples were collected around the farms, and sewage samples were collected from the river near the farms and downstream as well. The soil, dust, and sewage samples were collected at least in triplicate per farm. Tigecycline non-susceptible isolates were selected on MacConkey agar plates containing tigecycline (4 mg/L), and all plates were incubated at 37°C for 20 to 22 h. Then, one to three well-formed tigecycline non-susceptible colonies were randomly selected for polymerase chain reaction (PCR) screening of *tet*(X), and the *tet*(X4) subtypes were further confirmed by full-length amplification and Sanger sequencing, as previously described (Sun et al., 2019a). The bacterial species was identified by MOLDI-TOF MS Axima^TM^ (Shimadzu-Biotech Corp., Kyoto, Japan) and 16S rRNA sequencing. Then, for multiple isolates separated from the same sample, enterobacterial repetitive intergenic consensus (ERIC)–PCR was performed for preliminary typing using previous primers (Sun et al., 2019a), and different clones were kept.

### Antimicrobial Susceptibility Testing

Against the *tet*(X4)-positive strains, MICs of tigecycline, chlortetracycline, amikacin, gentamicin, meropenem, colistin, ceftazidime, cefotaxime, fosfomycin, ciprofloxacin, ampicillin, and sulfamethoxazole–trimethoprim were determined and interpreted according to the Clinical & Laboratory Standards Institute (CLSI) guidelines (CLSI, 2019). In particular, the breakpoint of colistin was interpreted in accordance with the EUCAST breakpoint (EUCAST, 2021). Florfenicol breakpoints were regarding the previous report (Michigan State University, 2014). *E. coli* ATCC 25922 served as the quality control strain.

### Whole-Genome Sequencing

Genomic DNA was extracted from *E. coli* strains using the Genomic DNA Purification Kit (Tiangen, China). DNA purity and concentration were determined using a NANODROP 2000c spectrophotometer. Whole-genome sequencing (WGS) was performed with the Illumina HiSeq 2500 system (Bionova Biotech Co., China) using the paired-end 2% 150-bp sequencing protocol. The draft genome was *de novo* assembled using SPAdes version 3.12.0. The putative coding sequences of the flanking regions of *tet*(X4) were obtained using RAST^1^. Multilocus sequence typing (MLST), plasmid in-compatibility (Inc) groups, antibiotic resistance genes (ARGs), and mobile elements were analyzed by Center for Genomic Epidemiology^2^ and ISfinder^3^.

### Molecular Typing

All *tet*(X4)-positive *E. coli* strains were classified by *Xba*I digested (Takara, Dalian, China) pulsed-field gel electrophoresis (PFGE) according to the PulseNet protocol^4^ using a CHEF Mapper System (Bio-Rad, Hercules, CA, United States). PFGE patterns were compared using BioNumerics version 6.6 (Applied Maths, Sint-Martens-Latem, Belgium) under appropriate optimization (1.5%) and tolerance (1.5%) settings and a cutoff at 85% similarity to delineate PFGE clusters. MLST was performed by the primers and protocol specified on the *E. coli* MLST database website^5^.

### Plasmid Characterization

The *tet*(X4) gene locations were identified using S1 nuclease-PFGE and Southern blot analysis. Briefly, DNA from donor strains and the transconjugants harboring *tet*(X4) were extracted and embedded in agarose gel plugs and then treated with S1 nuclease (Takara), and the DNA fragments were separated by PFGE. Southern blot hybridization was then performed with DNA probes specific for the *tet*(X4) gene that was non-radioactively labeled with a DIG High Prime DNA labeling and detection kit (Roche Diagnostics, Mannheim, Germany) (Versalovic et al., 1991).

### Conjugation and Transformation Analyses

To investigate the transferability of *tet*(X4)-bearing plasmid, conjugation assays were performed using streptomycin-resistance *E. coli* C600 as the recipient strain. Briefly, overnight cultures of donor and the recipient strains were 1:1 mixed and incubated at 37°C for 16 to 20 h. After incubation, 10-fold serial dilutions were mixed in sterile saline, and 100-μL samples were spread onto LB agar plates containing 4 mg/L tigecycline and 1,500 mg/L streptomycin. The *tet*(X4)-positive transconjugants were confirmed by PCR and ERIC-PCR (Versalovic et al., 1991; Sun et al., 2019b). Susceptibility of transconjugants was detected as mentioned previously. Plasmid analysis was performed using whole-genome sequence as described previously. Plasmid DNA was extracted using a Qiagen Prep Plasmid Midi Kit (Hilden, Germany).

## Results

With the detection ratio of 0.9%, 16 *E. coli* strains were identified harboring *tet*(X4) gene, among which nine were isolated from feces, six from soil, and one from sewage (Supplementary Table 1). The 16 *tet(X4)*-positive strains were detected from three provinces: Shandong, Jiangsu, and Guangdong (Figure 1).

**FIGURE 2:**
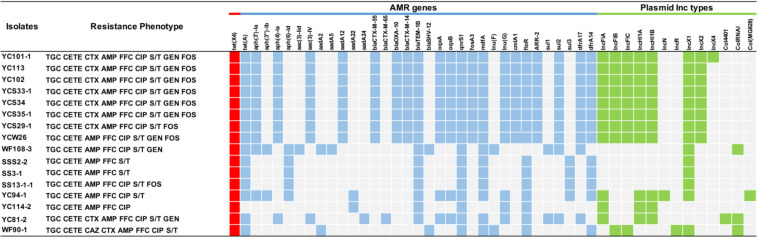
Antimicrobial resistance profiles of *tet*(X4)-positive *E. coli* isolates and the presence or lack of AMR genes are colored in blue or gray, respectively. The red squares indicate the presence of *tet*(X4) gene. The green squares indicate the plasmid type. TGC, tigecycline; CETE, chlortetracycline; MEM, meropenem; CAZ, ceftazidime; CTX, cefotaxime; AMP, ampicillin; CIP, ciprofloxacin; S/T, trimethoprim/sulfamethoxazole; GEN, gentamicin; AMK, amikacin; FOS, fosfomycin; CS, colistin. The breakpoint of CETE is not available, so resistance to CETE was defined regarding the resistant breakpoint of tetracycline (≥16 mg/L) (CLSI, 2019).

Antimicrobial susceptibility testing identified that all *tet*(X4)-positive *E. coli* strains were resistant to tigecycline showing MICs over 16 mg/L (8 mg/L of FDA breakpoint) (FDA, 2019). In addition, most of these strains were multiply resistant to ampicillin, chlortetracycline, florfenicol, fosfomycin, sulfamethoxazole-trimethoprim, and ciprofloxacin (Figure 2 and Supplementary Table 2). The MICs for colistin and meropenem for all *E. coli* strains were lower than 0.25 and 0.03 mg/L, respectively. In total, 9 of 16 isolates were resistant to gentamicin, with MICs ranging from 0.5 to 128 mg/L, whereas no isolates were resistant to amikacin (Supplementary Table 2).

**FIGURE 3:**
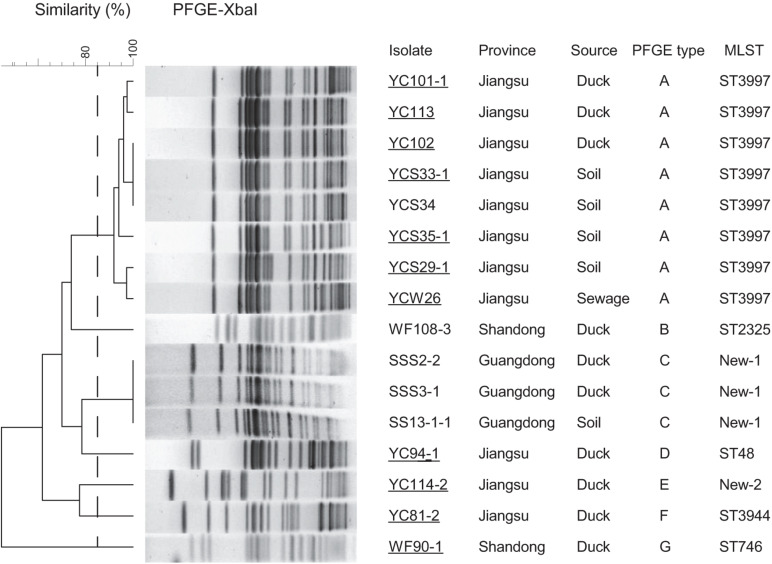
PFGE-X*bal* dendrogram and details about *tet*(X4)-positive *E. coli* isolates, PFGE patterns with a cutoff at 85% similarity were considered to be the same cluster and are included as groups A–G. Sampling details are listed in the right table, as well as the MLST analysis results of WGS. Conjugation assays to C600 succeeded for 11 isolates labeling with underline. Duck, duck feces.

We further characterized the molecular structures of the *tet*(X4)-positive isolates and *tet*(X4)-bearing plasmids. The distinct *tet*(X4)-positive isolates belonged to seven sequence types (STs): ST3997 (*n* = 8), ST2325 (*n* = 1), ST48 (*n* = 1), ST3944 (*n* = 1), and ST746 (*n* = 1) and another two new STs labeling as New-1 (*n* = 3) and New-2 (*n* = 1) in Figure 3. Interestingly, most isolates from Jiangsu province belonged to ST3997 sharing the same PFGE pattern suggesting clonal dissemination. Three *tet*(X4)-positive *E. coli* isolates from Guangdong province belonged to a new ST type (Figure 3).

In the mating tests, 11 *tet*(X4) genes were successfully transferred to *E. coli* C600, and S1-PFGE of both donors and transconjugants suggested that these genes were located on plasmids. The tigecycline MICs of the transconjugants were increased by at least 32-fold over the C600 recipient strains (Supplementary Table 2). Further, S1-nuclease digestion and Southern blot analysis showed that the *tet*(X4) genes were mostly located on plasmids in sizes of 200, 230, or 330 kb (Supplementary Figures 2A,B). Different incompatibility groups of IncHI1 and IncX1 plasmids carrying *tet*(X4) genes were confirmed by product enhanced reverse transcriptase testing of the corresponding transconjugants. During the mating assay, the cotransfer of IncHI1 and IncX1 plasmids was observed for YC101-1, YC113, YC102, and YCS29-1 as donors.

**FIGURE 4:**
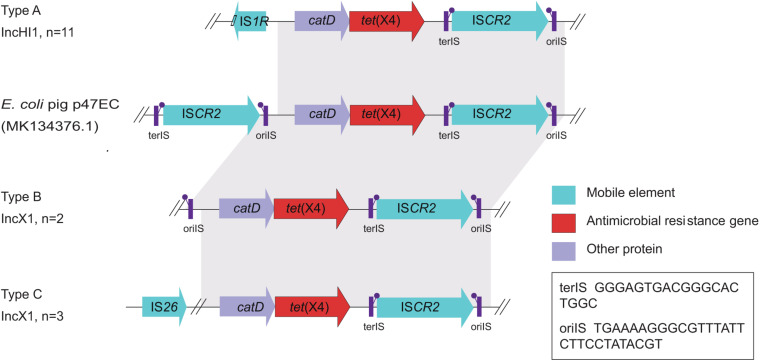
Gene alignments of the *tet*(X4) in isolates from this study and the reported plasmid p47EC. Mobile elements, antimicrobial resistance genes, and other proteins are shown as blue, red, and purple arrows, respectively. Regions of homology between 73 and 100% are marked by gray shading.

Moreover, WGS showed the *tet*(X4) gene was located primarily within three similar contigs in size of 2,700 to 9,553 bp bearing the core gene arrangement *cat*D-*tet*(X4)-*terl*S-IS*CR2-oril*S (Figure 4), which is identical to the reference plasmid p47EC (MK 134376.1) from *E. coli* (He et al., 2019). Mobile elements including △IS*1R* and IS*26* were identified at the upstream of *tet*(X4) gene in IncHI1 plasmid from 11 isolates and IncX1 plasmid from three isolates, respectively. This indicated that the *tet*(X4) genes are highly active and possibly will further transfer to other plasmids or isolates. For another two isolates, only *oril*S was found at the upstream of *tet*(X4) gene. Additionally, resistance genes of *tet*(A), *aph*, *aad*A, aadA2, *bla*_*CTX*_, *bla*_*TEM–1B*_, *bla*_*OXA*_, *bla*_*SHV*_, *oqx*AB, *qnr*S1, *fos*A3, *mdf*A, *lnu*, *erm*, *cml*A1, *flo*R, *sul*, and *dfr*A were detected in these *tet*(X4)-positive isolates as well (Figure 2).

## Discussion

For now, the *tet*(X4)-positive isolates were mostly reported in animals such as pigs, chickens, cows, and ducks. In our study, in the region of Southeast China, 0.7% (9/1211), 2.3% (6/259), and 0.4% (1/228) of studied fecal, soil, and sewage samples possessed *tet*(X4)-positive *E. coli* strains originated on duck farms. Although the detection ratio of *tet*(X4) in humans was low (0.07%), *tet*(X4) has been previously identified in pigs and chickens at slaughters, from soil and dust in animal farms (Sun et al., 2019a) (also in our study), and even pork from markets (Bai et al., 2019). Given the fact that tigecycline is not approved for veterinary use, the presence of *tet*(X4) in animals may be due to the high-level use of tetracycline derivatives for livestock (Bai et al., 2019). As one of the countries with the largest amount of antibiotics usage in the world, 12,000 tons (7.4%) of tetracyclines were consumed by China yearly (data from 2013), and most of them eventually entered into environment (Zhang et al., 2015). The *tet*(X4) gene possibly resulted from the evolution of *tet*X family members driven by the historical selective pressure due to the large-scale use of tetracyclines and had become the most worrisome resistant determinant to data. In addition, the importance of animals in the dissemination of tigecycline-resistance is only becoming recognized, and it might be just a matter of time when the *tet*(X4)-producing pathogens become widespread in public.

Whole-genome sequencing analysis showed that 16 *E. coli* strains belonged to seven different MLSTs (ST3997, ST2325, ST48, ST3944, and ST746 and another two new types). Among STs, ST3977 was the most prevalent type in Jiangsu province and has been reported harboring *bla*_*NDM–1*_ and *mcr-1* genes in animals (Novovic et al., 2016). ST48 has been identified in humans from various geographical areas (Nabal et al., 2019), indicating the wide spread of ST48 *E. coli* strains in public health. Multiple-drug resistance to chlortetracycline, ceftazidime, cefotaxime, ampicillin, florfenicol, ciprofloxacin, trimethoprim/sulfamethoxazole, and fosfomycin was observed in *tet*(X4) isolates. In accordance with the antimicrobial susceptibility tests, the WGS analysis indicated that more than 20 different ARGs were carried in these isolates. For instance, we identified *tet*(X4), *tet*(A), *aph*(3’), *aph*(4), *aph*(6), *aac*A, *bla*_*CTX*_, *bla*_*OXA*_, *bla*_*TEM*_, *oqx*AB, *qnr*S1, *fos*A3, *mdf*A, lnu(G), *flo*R, ARR-2, and *sul*2 genes in all the isolates from Jiangsu province. Notably, eight *E. coli* strains were detected cocarrying *bla*_*CTX–M–55*_ and *bla*_*CTX–M–14*_ genes, indicating the widespread and the diversity of ESBL resistance. Together with the similar PFGE patterns, it is possible that one epidemic clone has been disseminated in this region. The scenario is alarming as these isolates can act as an abundant reservoir spreading ARGs to both environment and humans.

We detected *tet*(X4) strains from both patterns A and C breeding system. Duck farming in pattern A system often has high density of animals, and the duck–fish production system (pattern C) is much more economical than most traditional crop farming and poultry husbandry systems, because it is based on the concept that “there is no waste,” and “waste is only a misplaced resource that can be a valuable input for other component” (Krag et al., 2015). There was no enough evidence in this study to demonstrate the connection of dissemination of *tet*(X4) with the different breeding patterns. However, it is concerning that among all the *tet*(X4)-positive isolates, 45% (5/11) of the samples were isolated from environments in Shandong and Jiangsu provinces. Additionally, AMR genes including *tet*(X4) were identified in isolates recovered from sewage, river, and soil of ponds in this study, indicating the possible dissemination of AMR genes in environment.

In conclusion, we identified 16 *tet*(X4)-positive *E. coli* isolates from duck farms in southeast China. Notably, this is the first study to report the development of diversity in the population of *tet*(X4)-positive *E. coli* isolates from ducks. WGS analysis further determined *tet*(X4) coexisted with other ARGs mediating multiple-drug resistance to chlortetracycline, ampicillin, florfenicol, ciprofloxacin, gentamicin, trimethoprim/sulfamethoxazole, and fosfomycin. The detection of *tet*(X4)-bearing IncHI1 and IncX1 plasmids in isolates from feces, soil, and even sewage samples enlarges our understanding of the dissemination of tigecycline-resistant genes as (i) *tet*(X4)-bearing IncHI1 and IncX1 plasmids were highly transferable and (ii) environmental-isolated strains could pose a greater threat to public health. It is absolutely the time to add the *tet*(X4) gene into the resistance surveillance.

## Data Availability Statement

The datasets presented in this study can be found in online repositories. The names of the repository/repositories and accession number(s) can be found below: NCBI SRA BioProject, accession no: PRJNA743422.

## Author Contributions

Y-HL, X-PL, and JS conceived of this study. YY designed the experiment and drafted the manuscript. YY and XK revised the manuscript. YY and C-YC analyzed the sequencing data. C-YC, CC, and XK performed the experiments. M-GW collected all the samples. All authors read and approved the final manuscript.

## Conflict of Interest

The authors declare that the research was conducted in the absence of any commercial or financial relationships that could be construed as a potential conflict of interest.

## Publisher’s Note

All claims expressed in this article are solely those of the authors and do not necessarily represent those of their affiliated organizations, or those of the publisher, the editors and the reviewers. Any product that may be evaluated in this article, or claim that may be made by its manufacturer, is not guaranteed or endorsed by the publisher.

## Footnotes

1
https://rast.nmpdr.org/

2
https://cge.cbs.dtu.dk/services/

3
https://www-is.biotoul.fr

4
http://www.pulsenetinternational.org/protocols/

5
http://enterobase.warwick.ac.uk
