# Supplementary material for: Prevalence of tet(X4) in Escherichia coli From Duck Farms in Southeast China
Source: Front Microbiol. 2021 Aug 23;12:716393. doi: 10.3389/fmicb.2021.716393 (PMC8419466; doi:10.3389/fmicb.2021.716393)
Supplement: Supplementary file 1 [file Data_Sheet_1.docx]

**APPENDIX.**

**Table S1.**Prevalence of *tet*(X4)-positivestrainsfrom fecal, soil, sewage, and dust samples in Southeast China.

| **Provinces** | **origin** | **Density/**  **area(m^2^)** | **FarmingSystem** | **Positive strains in fecal samples** | **Positive strains in soil samples** | **Positive strains in sewage**  **samples** | **Positive strains in dust samples** | **Positive strains in total samples** |
| --- | --- | --- | --- | --- | --- | --- | --- | --- |
| **Hainan** | Goose farm | 1000/3000 | C | 0/42 | 0/5 | 0/5 | 0/5 | 0/57 |
| **Guangdong** | Duck farm | 1200/3000 | C | 0/52 | 0/5 | 0/3 | 0/3 | 0/63 |
|  | Duck farm | 1500/2100 | C | 0/51 | 0/2 | 0/3 | 0 | 0/56 |
|  | Duck farm | 2000/2800 | C | 0/51 | 0/10 | 0/4 | 0/10 | 0/75 |
|  | Duck farm | 1500/4000 | C | 0/30 | 0/3 | 0/3 | 0 | 0/36 |
|  | Goose farm | 1000/3500 | C | 0/40 | 0/3 | 0/13 | 0 | 0/56 |
|  | Duck farm | 1000/4000 | C | 1/66 (1.5%) | 2/14(14.3%) | 0/15 | 0 | 3/95(3.2%) |
|  | Duck farm | 1500/4500 | C | 0/20 | 0/8 | 0/8 | 0 | 0/36 |
|  | Duck farm | 2000/3000 | C | 0/81 | 0/29 | 0/32 | 0 | 0/142 |
|  | Duck farm | 1500/4000 | C | 0/40 | 0/15 | 0/15 | 0 | 0/70 |
|  | Duck farm | 2000/4000 | C | 0/40 | 0/15 | 0/15 | 0 | 0/70 |
|  | Duck farm | 2000/4000 | C | 0/40 | 0/6 | 0/6 | 0 | 0/52 |
| **Guangxi** | Goose farm | 500/2000 | C | 0/25 | 0 | 0 | 0 | 0/25 |
|  | Duck farm | 5000/15000 | C | 0/80 | 0 | 0 | 0 | 0/80 |
| **Shandong** | Duck farm | 20000/2000 | A | 0/40 | 0/12 | 0/12 | 0 | 0/64 |
|  | Duck farm | 10000/1500 | A | 0/32 | 0/12 | 0/10 | 0 | 0/54 |
|  | Duck farm | 15000/2000 | A | 2/40 (5%) | 0/12 | 0/12 | 0 | 2/64 (3.1%) |
|  | Duck farm | 10000/2000 | A | 0/40 | 0 | 0 | 0 | 0/40 |
|  | Duck farm | 10000/2000 | A | 0/41 | 0 | 0 | 0 | 0/41 |
| **Fujian** | Duck farm | 3000/10000 | B | 0/40 | 0/12 | 0/12 | 0 | 0/64 |
|  | Duck farm | 1000/2500 | B | 0/40 | 0/12 | 0/12 | 0 | 0/64 |
|  | Duck farm | 1000/3000 | B | 0/40 | 0/12 | 0/12 | 0 | 0/64 |
| **Jiangsu** | Duck farm | 5000/16000 | B | 0/40 | 0/12 | 0/9 | 0 | 0/61 |
|  | Duck farm | 1500/4000 | B | 0/40 | 0/12 | 0/9 | 0 | 0/61 |
|  | Duck farm | 2000/4000 | B | 0/40 | 0/12 | 0/9 | 0 | 0/61 |
|  | Duck farm | 1000/2500 | B | 0/40 | 0/12 | 0/9 | 0 | 0/61 |
|  | Duck farm | 1500/3500 | B | 0/40 | 0/12 | 0/6 | 0 | 0/58 |
|  | Duck farm | 2000/4000 | A | 6/40 (15%) | 4/12 (33.3%) | 1/12 (8.3%) | 0 | 11/64 (17.2%) |
| **Total** |  |  |  | **9/1211 (0.7%)** | **6/259 (2.3%)** | **1/228 (0.4%)** | **0/18** | **16/1716 (0.9%)** |

Farming system, All duck and goose farms investigated in this study was divided into three different breeding patterns (Figure S2): A. on filter net and shelves; B. along the river without shed; C. duck-fish production system.

**Table S2.** MICs of thirteen antimicrobials agents against *tet*(X4)-positive isolates and transconjugants.

| **Isolate** | **MICs (mg/L)** | | | | | | | | | | | | |
| --- | --- | --- | --- | --- | --- | --- | --- | --- | --- | --- | --- | --- | --- |
|  | **TGC** | **CETE** | **MEM** | **CAZ** | **CTX** | **AMP** | **FFC** | **CIP** | **S/T** | **GEN** | **AMK** | **FOS** | **CS** |
| YC101-1 | 16 | 256 | 0.015 | 2 | 16 | >256 | 128 | 128 | 160 | 16 | 2 | >256 | 0.25 |
| YC113 | 16 | 128 | 0.008 | 2 | 16 | >256 | 128 | 128 | 160 | 16 | 2 | >256 | 0.25 |
| YC102 | 16 | 256 | 0.015 | 2 | 16 | >256 | 128 | 128 | 160 | 16 | 1 | >256 | 0.25 |
| YCS33-1 | 16 | 256 | 0.015 | 2 | 16 | >256 | 128 | 128 | 160 | 16 | 1 | >256 | 0.25 |
| YCS34 | 16 | 256 | 0.015 | 2 | 16 | >256 | 128 | 128 | 160 | 16 | 1 | >256 | 0.25 |
| YCS35-1 | 16 | 128 | 0.015 | 2 | 16 | >256 | 128 | 128 | 160 | 16 | 8 | >256 | 0.25 |
| YCS29-1 | 16 | 256 | 0.015 | 2 | 16 | >256 | 256 | 128 | 160 | 8 | 1 | >256 | 0.25 |
| YCW26 | 16 | 128 | 0.015 | 0.125 | 32 | 256 | 128 | 1 | 160 | 32 | 2 | >256 | 0.25 |
| WF108-3 | 32 | 256 | 0.03 | 8 | 2 | >256 | >256 | 64 | 160 | 128 | 1 | 4 | 0.25 |
| SSS2-2 | >64 | >256 | 0.015 | 0.125 | 0.06 | 256 | >256 | 1 | 160 | 1 | 2 | 2 | 0.25 |
| SSS3-1 | >64 | 256 | 0.015 | 2 | 0.06 | 256 | >256 | 1 | 160 | 1 | 2 | 2 | 0.25 |
| SS13-1-1 | 64 | 256 | 0.03 | 256 | 32 | 128 | 64 | 256 | 160 | 1 | 16 | 256 | 0.25 |
| YC94-1 | 32 | >256 | 0.015 | 0.25 | 0.125 | >256 | >256 | 32 | 160 | 0.5 | 1 | 4 | 0.25 |
| YC114-2 | 16 | 128 | 0.008 | 0.125 | 0.125 | 64 | 256 | 64 | 5 | 8 | 2 | 2 | 0.25 |
| YC81-2 | 64 | 256 | 0.015 | 2 | 64 | >256 | 256 | >256 | 160 | 1 | 1 | 4 | 0.25 |
| WF90-1 | 32 | 256 | 0.015 | 16 | 8 | >256 | >256 | 64 | 160 | 1 | 2 | 4 | 0.25 |
| ***Transconjugants*** | | | | | | | | | | | | | |
| YC101-1-T | 16 | 128 | 0.015 | 0.5 | 4 | >256 | 64 | 2 | 40 | 1 | 2 | 4 | 0.25 |
| YC113-T | 16 | 64 | 0.015 | 0.5 | 4 | 256 | 64 | 1 | 80 | 0.5 | 1 | 4 | 0.25 |
| YC102-T | 16 | 128 | 0.015 | 0.5 | 4 | 256 | 64 | 1 | 20 | 1 | 2 | 4 | 0.25 |
| YCS33-1-T | 16 | 128 | 0.015 | 0.5 | 8 | >256 | 128 | 2 | 160 | 32 | 2 | >256 | 0.25 |
| YCS35-1-T | 16 | 128 | 0.015 | 0.125 | 2 | 256 | 32 | 1 | 160 | 32 | 2 | >256 | 0.25 |
| YCS29-1-T | 16 | 128 | 0.015 | 0.25 | 2 | 256 | 128 | 1 | 160 | 32 | 2 | >256 | 0.25 |
| YCW26-T | 16 | 128 | 0.015 | 0.125 | 2 | 256 | 128 | 1 | 160 | 32 | 2 | >256 | 0.25 |
| YC94-1-T | 32 | 128 | 0.015 | 0.125 | 0.06 | 256 | 256 | 16 | 5 | 1 | 2 | 4 | 0.25 |
| YC114-2-T | 16 | 128 | 0.015 | 0.06 | 0.03 | 64 | 64 | 1 | 5 | 0.5 | 1 | 4 | 0.25 |
| YC81-2-T | 16 | 128 | 0.015 | 0.06 | 0.03 | 64 | 128 | 1 | 5 | 1 | 1 | 4 | 0.25 |
| WF90-1-T | 32 | 128 | 0.015 | 4 | 2 | 256 | 128 | 2 | 160 | 1 | 2 | 4 | 0.25 |
| ***Recipient*** |  |  |  |  |  |  |  |  |  |  |  |  |  |
| *E. coli*C600  C | 0.5 | 4 | 0.015 | 0.06 | 0.03 | 2 | 1 | 0.03 | 5 | 2 | 1 | 4 | 0.125 |
| ***Resistance***  ***Breakpoint*** | ≥8^c^ | NA | ≥4^a^ | ≥16^a^ | ≥4^a^ | ≥8^b^ | 8^d^ | ≥1^a^ | ≥76/4^a^ | ≥16^a^ | ≥64^a^ | ≥256^a^ | ≥2^b^ |

TGC, tigecycline; CETE, chlortetracycline; MEM, meropenem; CAZ, ceftazidime; CTX, cefotaxime; AMP, ampicillin; FFC, florfenicol;CIP, ciprofloxacin; S/T, trimethoprim/sulfamethoxazole; GEN, gentamicin; AMK, amikacin; FOS, fosfomycin; CS, colistin. NA, not available.The breakpoint of CETE is not available, so resistance to CETE was defined regarding the resistant breakpoint of tetracycline (≥16 mg/L) (CLSI, 2019). a, breakpoints from CLSI (CLSI 2019); b, breakpoints from EUCAST 2021 (EUCAST 2021); c, breakpoints from FDA (FDA, 2019, https://www.fda.gov/drugs/development-resources/tigecycline-injection-products); d, breakpoints for florfenicol regarding the previous reports (Michigan State University DCPAH, 2014).

Michigan State University DCPAH (2014). Resistance Breakpoints for Antimicrobials Used in Animals. 1–8.


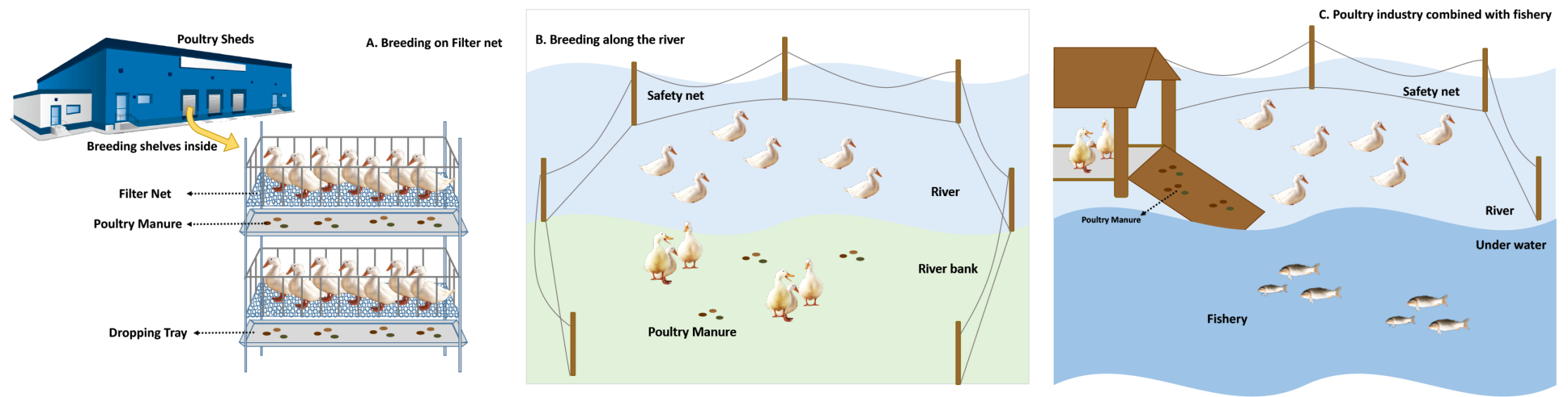


**Figure S1.** Three breeding patterns of duck and goose farms in Southeast China in this study.

**Figure S2a.** PFGE analysis (right) of *tet*(X4)-producing strains and corresponding transconjugants digested with S1 nuclease and hybridization with *tet*(X4) gene probe (left). Red arrows showed the location and size of *tet*(X4)-carrying plasmids.

**Figure S2b.** PFGE analysis (right) of *tet*(X4)-producing strains and corresponding transconjugants digested with S1 nuclease and hybridization with *tet*(X4) gene probe (left). Red arrows showed the location and size of *tet*(X4)-carrying plasmids.
